# Thromboembolic Antiphospholipid Syndrome (APS): Efficacy and Safety of Different Anticoagulants-Results of the APSantiCO Registry

**DOI:** 10.3390/jcm11164845

**Published:** 2022-08-18

**Authors:** Annabel Schulz, Eva Herrmann, Olivia Ott, Edelgard Lindhoff-Last

**Affiliations:** 1 Coagulation Centre, Cardiology Angiology Centre Bethanien Hospital (CCB), 60389 Frankfurt, Germany; 2 Coagulation Research Centre Bethanien Hospital, 60389 Frankfurt, Germany; 3 Institute of Biostatistics and Mathematical Modelling, Goethe University, 60590 Frankfurt, Germany

**Keywords:** antiphospholipid syndrome, anticoagulation, direct oral anticoagulants, arterial thrombosis, vitamin K antagonists, anti-phospholipid antibodies, venous thrombosis

## Abstract

**Background:** The particular challenge in dealing with patients with thromboembolic antiphospholipid syndrome (APS) is to establish an adequate therapy regime, as patients suffer from an increased risk of relapse despite antithrombotic treatment (ATT). Vitamin K antagonists (VKA) are the standard medication of choice. The current data on the use of direct oral anticoagulants (DOAC) in APS patients remain limited. **Methods:** The results of the retrospective APSantiCO registry are presented. In 80 patients with APS, the efficacy and safety of different ATT regimens were analyzed. **Results:** At the time of inclusion, 43.8% of patients were treated with VKA and 36.3% with DOAC. Medication regimes changed several times and 279 treatment phases were further analyzed with a total treatment length of 7529 months. The incidence of recurrent arterial thrombosis was significantly larger in the DOAC group compared with the VKA group (*p* < 0.001), while the incidence of recurrent venous thrombosis was comparable between both groups, as was the incidence of bleedings. Heavy menstrual bleeding was the most frequently observed bleeding complication. **Conclusions:** The data suggest that DOAC may be an alternative to VKA for APS patients with venous thromboembolism, while VKA should be used in APS-related arterial thrombosis.

## 1. Introduction

Antiphospholipid syndrome is an acquired thrombophilic diathesis, which can occur either in its primary form (primary APS) or in association with other autoimmune diseases, such as lupus erythematosus [1], so-called secondary APS [2].

According to the Sydney Consensus Statement criteria [2], diagnosing antiphospholipid syndrome (APS) depends on the existence of at least one clinical criterium, such as a vascular thrombosis (arterial, venous or small vessel thrombosis) and/or pregnancy morbidity, and the presence of laboratory criteria, such as the presence of lupus anticoagulants (LA) and/or antibodies directed against ß2-glycoprotein I (ß2GPI) and/or anticardiolipin antibodies (aCL), on a minimum of two occasions, at least 12 weeks apart.

Existing study results suggest that long-term oral anticoagulation therapy after a thromboembolic event is beneficial in APS patients [3]. Some authors, as well as the European League against Rheumatism (EULAR) recommendations for the management of antiphospholipid syndrome in adults, recommend vitamin K antagonists (VKA) as treatment for APS patients [4]. The drugs are used to prevent recurrent thrombosis with an advised target INR in a range of 2.0 to 3.0 [5,6]. This treatment option should be chosen especially for high-risk patients with a triple-positive antibody profile or with the presence of LA [7].

The TRAPS Trial, which investigated the efficacy and safety of rivaroxaban in comparison to VKA in high-risk APS patients with triple-antibody positivity, suggested inferiority in rivaroxaban in protecting against recurrence of arterial thrombosis and, therefore, was terminated prematurely [8]. These data were responsible for a recommendation by the European Medicines Agency (EMA) that DOAC should be avoided in patients with APS, especially in those with a triple-positive-antiphospholipid-antibody (aPL) profile [9]. These EMA recommendations were not exclusively limited to the patient population with triple positivity, but were extended to any APS patient [10]. Therefore, at present, treatment with DOAC in APS patients might be a use against professional advice.

In contrast, some experts’ statements and studies suggest that the use of DOAC in a specific APS patient population may be beneficial and not inferior in efficacy and safety compared to VKA [7,11,12,13,14].

Therefore, the role of DOAC in patients with thromboembolic APS remains uncertain due to the limited clinical data and the low number of prospective randomized trials due to the rarity of this disease. Further, the patient clientele is not easy to diagnose due to its heterogeneity and its individual risk profile, depending on both medical history and fluctuating aPL-antibody profiles.

Through retrospective analysis of different antithrombotic treatment regimens, including DOAC, in a cohort of well-defined APS patients treated between 2015 and 2021, the efficacy and safety of different types of anticoagulant treatments were analyzed to increase knowledge and clinical experience on treatment with DOAC in clinical routine.

## 2. Materials and Methods

The APSantiCO registry was generated by retrospectively reviewing all medical records of APS patients treated in the center of Thrombosis and Hemostasis at the Bethanien hospital, Frankfurt, Germany, between January 2015 and October 2021. For this purpose, we used the keyword searching option of the electronic patient database, focusing on the term “Antiphospholipid syndrome”. In addition, continuous screening of suitable newly presented patients during inpatient and outpatient consultations was performed.

According to the search query, a list containing 135 patient IDs was received, which was used as a basis for further analyses. Further, 7 patients were additionally included in the study from current consultations.

We included all APS patients, who matched the Sydney classification criteria for APS [2] and also met the following criteria:

- Minimum age of 18 years;
- The presence of any APS antibody risk profile (single/double/triple positivity);
- Arterial and/or venous thromboembolism.

**Figure 1:**
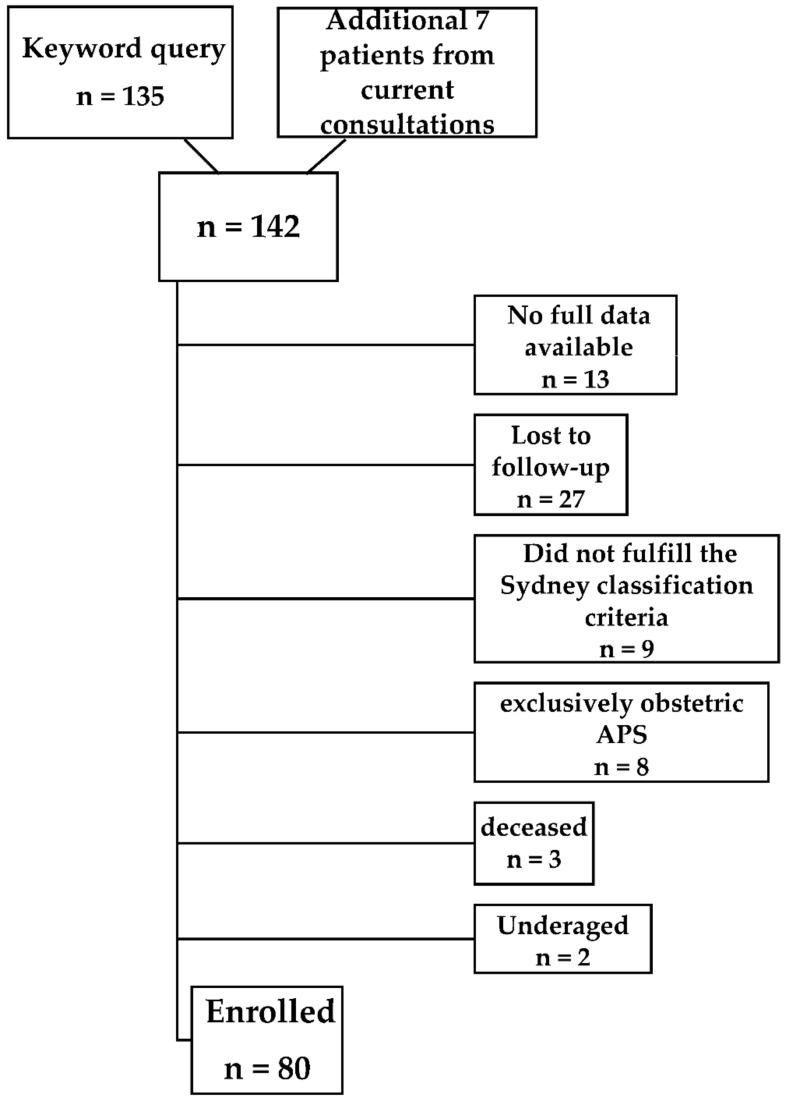
Flowchart of patient inclusion.

We excluded patients without anticoagulant medication and female patients exclusively suffering from obstetric APS without thrombosis. Furthermore, we ruled out underaged patients or patients who had already died but were still registered in our patient database, as well as patients with inadequately diagnosed APS. We finally identified 80 patients who fulfilled the Sydney classification criteria for APS (see Figure 1).

We retrospectively collected the following clinical data using electronic patient records: gender, current age, as well as age at the first vascular event, comorbidities, medical history, cardiovascular risk factors, and aPL profiles.

For detection of anti-β2 glycoprotein IgG and IgM and anticardiolipin IgG and IgM a fully automated chemiluminescence immunoassay (Hemosil^®^ AcuStar Anti-Cardiolipin IgG and IgM; Hemosil^®^ AcuStar Anti-β2-Glycoprotein-I IgG and IgM, Instrumentation Laboratory, Bedford, MA, USA) was used.

For detection of lupus anticoagulants, diluted Russels Viper Venom Time assays (dRVVT, Hemosil^®^ dRVVT Screen and Hemosil^®^ dRVVT Confirm, Instrumentation Laboratory, Bedford, MA, USA) were used. In addition we used a lupus-sensitive aPTT-based assay (Mixcon LA-test, Instrumentation Laboratory, Kirchheim, Germany), described before [15].

To differentiate between primary and secondary APS, we collected data on the occurrence of autoimmune diseases including systemic lupus erythematosus (SLE), sharp syndrome, Sjögren’s syndrome, etc. In addition, we analyzed the previous medication history before the patient’s first visit at our department whenever possible by contacting the responsible general practitioners and the hospitals in case of inpatient treatments. Focusing on our study-specific research questions we predominantly collected data concerning recurrent venous and/or arterial thrombotic events while on any kind of antithrombotic medication and events of major bleeding (MB) [16] and clinically relevant non-major bleeding (CRNMB) defined as per ISTH criteria [17]. All data used for this evaluation were taken from the patient’s medical records and were updated and completed by telephone interviews as part of the routine quality assurance process.

The registry is listed in Trials.gov: NCT05195372 and ethical approval was obtained from the local Ethics Committee (approval number 2021-2520-evBO).

### Statistical Analysis

The main observational outcome measure was the occurrence of recurrent thromboembolic events (arterial and/or venous) during therapy with DOAC compared to VKA and/or other anticoagulant medication regimens in APS patients. Furthermore, we investigated the occurrence of hemorrhagic complications under the respective therapeutic agents.

Group comparisons for qualitative or quantitative data were performed using two-sided binominal tests, Fisher’s exact test and Wilcoxon–Mann–Whitney test, respectively.

Data are summarized by counts (percentages) and median with range (25–75% percentile). Incidence rates were described as rates per 100 patient years and 95% confidence intervals. Incidence risk ratios were estimated by a mixed-effect negative binomial regression to account for dependence of incidences in different treatment phases within single patients. For statistical analysis R software version 4.1.0 (R Foundation for Statistical Computing, Vienna, Austria) with the packages lme4 and MASS was used.

## 3. Results

### 3.1. Patient Characteristics

Eighty patients fulfilled the inclusion criteria and were included in the retrospective analysis. Demographic data were analyzed depending on their first index event (see Table 1). We found that 73.8% (59/80) of patients presented with primary APS and 25% (20/80) of patients had a secondary APS, including 10 patients whose APS was associated with systemic lupus erythematosus (SLE). One patient could not be clearly assigned to any group. Gender was distributed equally (52.5% women and 47.5% men), after all female patients without thrombosis and with sole pregnancy-related complications were excluded. The median age at index event was 44.5 years (25–75% percentiles: 30.8–59.3 years) and the median BMI was 26.3 kg/m^2^ (25–75% percentiles: 24.2–29.9 kg/m^2^). Further, 71.3% (57/80) of patients initially presented with a venous thromboembolism, including deep leg vein thrombosis, pulmonary embolism, superficial vein thrombosis, and atypical venous thrombosis (for more details, see Table 1). Moreover, 28.8% (23/80) of patients had an arterial index event. The majority of these events (52.2%; 12/23) was caused by strokes or TIAs, followed by myocardial infarction (26.1%; 6/23), peripheral artery occlusions (13%; 3/23), and atypical arterial embolisms (8.7%; 2/23).

The majority of patients presented with cardiovascular risk factors (86.3%, 69/80), including hypercholesterolemia (52.5%), hypertension (48.8%), smoking (37.5%), elevated lipoprotein a (13.8%), and diabetes mellitus (8.8%). Autoimmune diseases were observed in 28.8% of the investigated patients and 11.3% had an inherited thrombophilia.

At the time of inclusion, 33 patients were “triple positive” (41.3%), 25 were “double positive” (31.3%) and 22 patients (27.5%) had only one type of aPL, including 10 patients (12.5%) with single positivity for LA (see Table 1). The majority of triple-positive patients were treated with VKA (60.6%; 20/33), while patients treated with DOAC had lower-risk APA profiles.

At inclusion, 36.3% (29/80) patients were treated with a DOAC (3 patients with rivaroxaban, 25 patients with apixaban, and 1 patient with edoxaban) and 43.8% (35/80) with a VKA. During data collection, it became apparent that the medication regime changed several times within a patient’s life due to various influences, such as relapse events, drug intolerance, family planning, or complications caused by the medication. In total, 279 treatment phases in 80 patients with a total length of 7529 months were used as a basis for further statistical analysis of recurrent thrombosis and bleeding events. Further, 62.5% of patients (50/80) received at least one treatment phase with a DOAC. In total, 88 DOAC-treatment phases were analyzed (26 with Rivaroxaban, 50 with Apixaban, 7 with edoxaban, 5 with dabigatran) and 58.8% of patients (47/80) received VKA at one or more time periods. In total, 83 VKA-treatment phases were further analyzed. The total length of treatment was 1575 months in DOAC-treated patients and 3368 months in VKA-treated patients. Further, 62.5% of patients (50/80) were treated at one or more time points with s.c. anticoagulation and/or platelet inhibitors, resulting in 108 treatment phases. In this patient group, the total length of treatment was 2586 months.

By file reviews and follow-up examinations, 65 recurrent thromboembolic events, despite anticoagulation, were recorded, 31 arterial thrombosis (6 events during VKA-treatment, 12 events during DOAC treatment, and 13 events during treatment with parenteral anticoagulants and/or aggregation inhibitors), and 34 venous thrombosis (10 events during VKA treatment, 5 during DOAC treatment, and 19 events during treatment with parenteral anticoagulants and/or aggregation inhibitors). Both arterial and venous recurrent thrombosis occurred in five APS patients.

### 3.2. Recurrent Arterial Thrombosis

**Figure 2:**
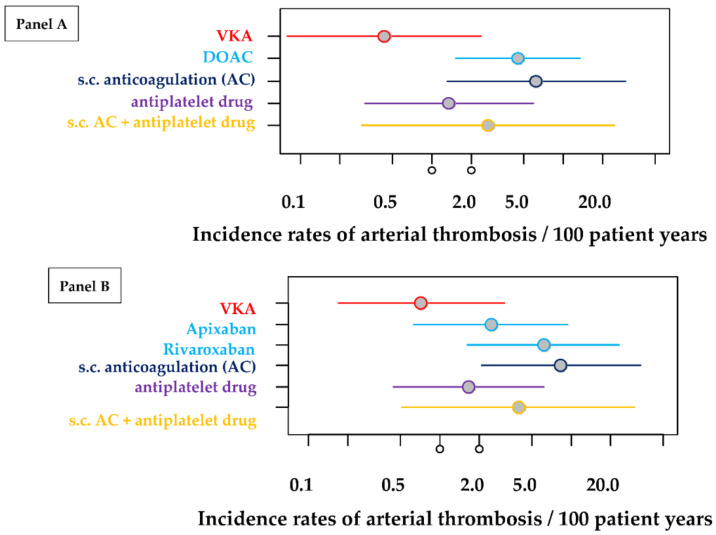
Incidence rates of arterial thrombosis per 100 patient years for different treatments together with 95% confidence intervals. The estimations of these rates are based on 279 treatment periods of 80 patients. (Panel (**A**)) estimations of all treatment periods by pooling all DOAC patients. (Panel (**B**)) estimations of treatment periods differentiating between the different types of DOAC (rivaroxaban and apixaban) but excluding data from some treatment periods with dabigatran and edoxaban.

We found that 31 arterial events, despite antithrombotic therapy, occurred in 19 patients. Ischemic strokes or TIAs were most frequently observed (61.3%; 19/31), followed by peripheral arterial occlusions (19.4%; 6/31). Atypical arterial embolisms were observed in four patients and myocardial infarction in two cases. Incidence rates of arterial thrombosis per 100 patient years for different treatments were calculated and, in addition, incidence risk ratios using treatment with VKA (“gold standard”) as reference. Results are given in Table 2 and in Figure 2A,B.

As there were only a few incidences overall, estimators have an inherent inaccuracy and the absolute rates and ratios have to be interpreted with care. The incidence of arterial thrombosis was significantly larger in the DOAC group compared with the VKA group (*p* < 0.001; Table 2, Figure 2A). Because of the estimation variability, other estimates also slightly differ. In a second modelling approach, the analysis was repeated, differentiating between the two largest DOAC groups (rivaroxaban and apixaban). For reasons of clarity, the results of the second evaluations were combined in one table (see Table 2). Here, the enhancement of the incidence risk is smaller for treatment with apixaban compared to treatment with rivaroxaban and only significantly enhanced for treatment with rivaroxaban when compared with VKA treatment as reference (apixaban: *p* = 0.1518, rivaroxaban: *p* = 0.0058).

For the purpose of visualization, Figure 2A,B were generated from the incidences that occurred (per 100 patient years). Figure 2A shows the incidences in relation to 100 patient years, taking into account the observation arm of subjects taking DOAC. Figure 2B, on the other hand, differentiates further between apixaban and rivaroxaban.

As can be seen from the graph, the value of the incidences that occurred when the gold-standard VKA was used is the lowest, followed by antiplatelet inhibition and antiplatelet inhibition in combination with s.c. anticoagulation. Incidences for s.c. administered anticoagulation alone and DOAC are similar. Differentiating DOACs into apixaban and rivaroxaban, there is a slight advantage when apixaban is used. Nevertheless, there are wide confidence intervals (CI) and a failure to reach a *p*-value of alpha < 0.05 (only with DOAC, s.c. anticoagulation and Rivaroxaban, *p* values are <0.05).

### 3.3. Recurrent Venous Thrombosis

Recurrent venous thromboses occurred in a similarly high number of cases (34 in total in 21 patients) as arterial recurrences (31 in total in 19 patients).

**Figure 3:**
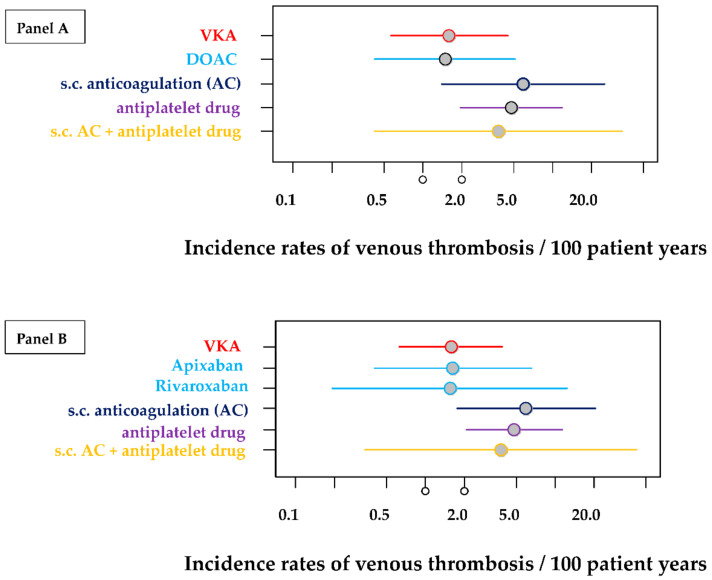
Incidence rates of venous thrombosis per 100 patient years for different treatments together with 95% confidence intervals. The estimations of these rates are based on 279 treatment periods of 80 patients. (Panel (**A**)) estimations of all treatment periods by pooling all DOAC patients. (Panel (**B**)) estimations of treatment periods differentiating between the different types of DOAC (rivaroxaban and apixaban) but excluding data from some few treatment periods with dabigatran and edoxaban.

Incidence rates of venous thrombosis per 100 patient years for different treatments were calculated and, in addition, incidence risk ratios using treatment with VKA (“gold standard”) as reference. Results are given in Table 3 and in Figure 3A,B.

The incidence of recurrent venous thrombosis was comparable between the DOAC and VKA groups (see Table 3, Figure 3A). This was also confirmed in a second modelling approach, differentiating between the two largest DOAC groups (rivaroxaban, apixaban). Incidences of venous thrombosis were quite comparable between the VKA, rivaroxaban, and apixaban groups (see Table 3, Figure 3B).

While DOAC and VKA are almost equally protective for recurrent venous thromboembolism (measured by the incidence rate), antiplatelet inhibitors show inadequate protection, with incidence rates as high as 4.8 (95% CI: 1.9–11.7). The use of UFH, LMWH, or Fondaparinux (s.c. anticoagulation) alone also shows a higher incidence rate (5.9; 95% CI: 1.4–25.2). Even the combination of antiplatelet inhibition and subcutaneous anticoagulation shows a higher incidence of venous recurrence (3.8; 95% CI: 0.4–34.5). Again, there are wide confidence intervals. It is important to mention that almost none of the calculated results can be considered significant, as the *p*-value reaches the significance level of alpha < 0.05 only in the case of antiplatelet inhibition.

Figure 3A,B clarify the information in Table 3. It can be seen that VKA and DOAC (Figure 3A) have virtually the same contact area and should, therefore, protect against venous thrombosis similarly well.

The analysis in Figure 3B allows a differentiated assessment of apixaban and rivaroxaban. Although rivaroxaban appears to have a minimal advantage, evident from the leftward shift, this assumption is diminished by rivaroxaban’s wide confidence interval.

### 3.4. Bleeding Events (MB and CRNMB)

**Figure 4:**
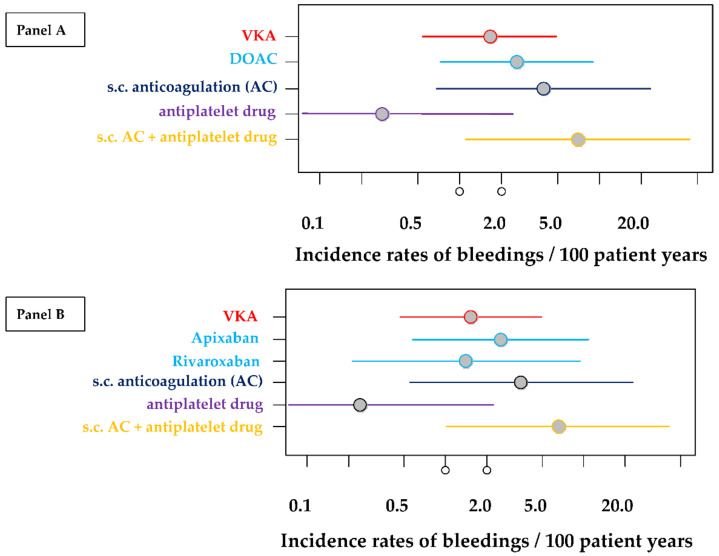
Incidence rates of bleedings per 100 patient years for different treatments together with 95% confidence intervals. The estimations of these rates are based on 279 treatment periods of 80 patients. (Panel (**A**)) estimations of all treatment periods by pooling all DOAC patients. (Panel (**B**)) estimations of treatment periods differentiating between the different types of DOAC (rivaroxaban and apixaban) but excluding data from some few treatment periods with dabigatran and edoxaban.

Incidence rates of bleeding events (MB and CRNMB) per 100 patient years for different treatments were calculated and, in addition, incidence risk ratios using treatment with VKA (“gold standard”) as reference. Results are given in Table 4 and in Figure 4A,B.

The incidence of bleedings is highly comparable between all different treatment groups and there are no significant differences when compared with VKA treatment (see Table 4, Figure 4 panel A). This is also confirmed in the more detailed analysis and neither apixaban treatment nor rivaroxaban treatment showed significant differences when compared with VKA treatment (Table 4, Figure 4B). None of the calculations reached the significance level of alpha < 0.05.

In total, 21.3% (17/80) of patients developed clinically relevant bleeding complications during different treatment regimes, including 70.6% (12/17) of female patients. The 17 affected registry participants showed a total of 23 bleeding events, as four female patients and one male patient developed bleeding complications more than once.

Eight clinically relevant bleeding events occurred during treatment with VKA, including three MB (one hematoma, one gastrointestinal bleeding: GIB, one subdural hematoma) and five CRNMB (two cases of menorrhagia, one GIB, one postoperative bleeding, one muscle hemorrhage). During treatment with DOAC, eight clinically relevant bleeding complications were observed, four MB (three cases of menorrhagia, one GIB), and four CRNMB (one hematoma, one spontaneous bleeding into the eyelid, one meniscus hemorrhage, and one patient with hematochezia and hemoptysis). During treatment with platelet aggregation inhibitors and/or subcutaneous anticoagulation, seven bleeding events occurred, four MB (two cases of hematoma, one menorrhagia, and one cerebral hemorrhage) and three CRNMB (one GIB, one patient with massive petechia, and one case of severe haematoma). Severe menorrhagia (50%; 6/12, only related to women) was the most common bleeding complication observed (26.1%, 6/23), followed by severe hematoma (21.7%, 5/23). Two intracranial hemorrhages occurred: one subdural hematoma under VKA and one cerebral hemorrhage under the combination therapy of LMWH and platelet inhibition.

Table 4 and Figure 4A,B show that the lowest incidence of bleeding is observed in the platelet-inhibitor treatment group but none of the calculations reached the significance level of alpha < 0.05. Combination therapy consisting of antiplatelet inhibition plus subcutaneously applied anticoagulants seems to be particularly prone to bleeding complications, with an incidence rate of 6.9 (95% CI: 1.1–43.5). However, it is important to note the extremely wide confidence interval, which is due to the short observation and treatment period, as only very few treatment phases have been described with this therapy regime.

## 4. Discussion

Adequate treatment of antiphospholipid syndrome remains difficult due to recurrent thromboembolic events, despite the proven efficacy of anticoagulant therapy. The many changes of drugs and drug classes in the APSantiCO registry show that adequate drug therapy in the APS patient population can prove to be very difficult and must be understood as a dynamic process, as the patient’s treatment is affected by various influencing factors. Especially in the case of a relapse, despite therapy with anticoagulants and/or antiplatelet agents, it is important to react quickly by optimizing or even changing the current medication. Therefore, it is often left to the treating physician to decide on a suitable therapy concept. Even with the “gold standard” VKA, recurrences still occur or bleeding complications and side effects against the use of VKA arise that require an alternative treatment strategy.

### 4.1. Recurrent Arterial and Venous Thrombosis

The results of the APSantiCO registry show that—in everyday clinical practice—DOAC may not adequately protect against recurrent arterial thrombosis, whereas VKA can. This is in agreement with the study results of the recently published TRAPS study. The TRAPS Trial (Trial on rivaroxaban in antiphospholipid syndrome), a prospective, non-inferiority, multicenter randomized controlled trial (RTC), with blinded end point adjudication, compared VKA (Warfarin) to rivaroxaban 15–20 mg once daily, regarding efficacy and safety in a high-risk triple-positive APS patient population [8]. As such, 120 APS patients (DOAC: *n* = 59, VKA: *n* = 61) were included and the trial was terminated prematurely due to seven arterial events in the rivaroxaban arm compared to no arterial events in the VKA arm.

In contrast, the results of the APSantiCO registry show that DOAC was similarly protective against venous recurrent events as VKA in predominantly low-risk patients. This is in agreement with a recently published retrospective study conducted by Liu et al. [18]. Further, 143 single-positive APS patients were included and examined with regard to the risk of recurrent thromboembolic events. As such, 64% of the study participants received VKAs, while 36% were treated with a DOAC (apixaban or rivaroxaban). After a median follow-up of 54 months, recurrent events occurred in 6.6% of patients receiving VKA and in 5.8% of patients receiving DOAC, which were predominantly venous recurrences. This study suggests that the use of DOAC may be considered as a safe alternative to VKA in selected APS patient populations with single-positive antibody status [18]. In this registry, a large proportion of patients treated with DOAC developed a venous thromboembolism as an initial event. A second recently published retrospective study, conducted by Kwan et al., investigated the risk of recurrent thromboembolism in 50 non-triple-positive APS patients without a history of arterial thromboembolism who were treated with DOAC at two tertiary care hospitals from January 2010 to July 2020. Outcomes of recurrent venous and/or arterial thromboembolism were assessed analyzing 157.2 years of patient follow-up. There were no recurrent VTEs, but one patient had a possible arterial thrombosis (0.64 events per 100 patient years (95% confidence interval [CI: 0.16–35.49])) as a transient ischemic attack (TIA), which occurred on reduced-dose DOAC. This study also concluded that DOAC may be an effective and safe treatment option for low-risk APS patient populations with venous thrombosis [19].

Malec et al. performed a prospective cohort study, comparing the efficacy and safety of DOAC in comparison to VKA in 176 APS patients, including 82 subjects who preferred DOAC or had unstable anticoagulation with VKA. The mean follow-up time was 51 months [20]. APS patients treated with DOAC had an increased risk of recurrent thromboembolic events and recurrent VTE alone compared with those on VKAs (hazard ratio (HR) = 3.98, 95% confidence interval (CI): 1.54–10.28, *p* = 0.004 and HR = 3.69, 95% CI: 1.27–10.68, *p* = 0.016, respectively) with no differences between rivaroxaban and apixaban or single- or double-positive and triple-positive APS. Thromboembolism in DOAC-treated patients was associated with older age (median 52 versus 42 years, *p* = 0.008). They hypothesized that the failure of DOAC in their study might have been related to poor compliance and insufficient knowledge of DOAC because 40% of the DOAC-treated patients who developed thromboembolism reported interrupted anticoagulation.

Williams et al. conducted a single-center retrospective cohort study in APS patients with single- or double-antibody positivity and a history of venous thrombosis. Among 96 patients included, 57 were prescribed warfarin and 39 received a DOAC (90% rivaroxaban). The proportion of patients with a recurrent thromboembolism was almost three-times higher in the DOAC group (15.4%) compared to the warfarin group (5.3%), although this was not statistically significant (*p* = 0.15). They concluded that their findings suggest that rivaroxaban may pose an increased risk for recurrent thromboembolism, even in low-risk APS patients compared to warfarin [21].

In a single-center prospective cohort study performed by Martinelli et al., including 28 patients diagnosed with APS, 15 patients were treated with VKA and 13 received rivaroxaban. In the two treatment arms, almost 50% of the patients had all three antibody classes and were, thus, classified as high-risk (triple-positive) subjects. The reported five relapses, despite medication, occurred exclusively in this triple-positive patient group. During treatment with VKA, recurrences occurred at an incidence rate of 2.4 (95% CI: 0.2–11.3) per 100 patient years (one event). In patients taking rivaroxaban, there were four events reported, causing an incidence rate of 19.4 per 100 patient years (95% CI: 6.5–46.2). All patients were triple positive for aPL antibodies [22]. Four events were of arterial origin (three acute myocardial infarction; one stroke) and one was venous (cerebral vein thrombosis), while no bleeding complications were reported. Here, like in the APSantiCO registry, mainly arterial recurrences were observed during treatment with DOAC.

The multicenter prospective randomized open-label study ASTRO-APS, launched by Woller et al., assigned anticoagulated patients with thrombotic APS to apixaban or warfarin (target international normalized ratio 2–3) and investigated the incidence of clinically overt thrombosis and vascular death for a period of 12 months. Between February 2015 and March 2019, 23 patients were randomized to apixaban and 25 to VKA. The study protocol was modified several times as increasing the apixaban dose from initially 2.5 mg twice daily to 5 mg twice daily and excluding APS patients with arterial thromboembolism. Among the components of the primary efficacy outcome, only stroke occurred in 6 of 23 patients, randomized to apixaban, compared to 0 of 25 patients randomized to warfarin. Finally, the study was terminated prematurely due to the slow recruitment rate and the clustered occurrence of strokes under apixaban. Since multiple protocol changes were carried out during the study and the number of patients included is small (48, instead of 200 estimated patients), conclusions are only possible to a limited extent [23].

Randomized clinical trials comparing DOAC with VKA in this heterogenous patient population are needed. Results of ongoing studies, such as the RISPAPS (rivaroxaban for stroke patients with antiphospholipid syndrome) phase 2/3 RCT, which aims to assess the efficacy of high-intensity rivaroxaban 15 mg twice daily versus high-intensity warfarin in patients diagnosed with APS and stroke or other brain ischemic injury, are urgently awaited (clincalTrials.gov Identifier: NCT03684564).

### 4.2. Bleeding Complications (MB and CRNMB)

The frequency of bleeding complications in the APSantiCO registry was similar for VKA and DOAC. Interestingly, the most frequently observed bleeding complication was severe menorrhagia followed by severe hematoma. This contrasts with the TRAPS trial, in which more bleeding events were observed in the rivaroxaban arm (*n* = 4) than in the warfarin arm (*n* = 2) [8]. In the retrospective trial conducted by Kwan et al., no major bleeding was observed but two patients had CRNMB (1.27 events per 100 patient years (95% CI: 1.5–46.0)), both as menorrhagia [19]. In the prospective study of Malec et al., patients on DOAC had an increased risk of MB or CRNMB (HR = 3.63, 95% CI: 1.53–8.63, *p* = 0.003), but rates of gastrointestinal bleeds (HR = 3.36, 95% CI: 0.70–16.16, *p* = 0.13) and major bleeds or CRNMB, other than heavy menstrual bleeding (HR = 2.45, 95% CI: 0.62–9.69, *p* = 0.2), were similar in both treatment groups [20].

### 4.3. Limitations

While generating the APSantiCO registry, we became aware of the extremely heterogeneous nature of APS presentation and treatment. To depict the real daily clinical practice, we decided to not only focus on DOAC in comparison to VKA but to include all types of antithrombotic regimes. Because we wanted to represent a very broad spectrum of anticoagulation therapies without excluding any options, we worked with small subgroups, which are hard to use in statistical analyses and, therefore, often yield results that are not significant. It should be mentioned that during the search of the literature, we noticed that randomized clinical trials often exclude patients with a history of recurrent thrombosis during anticoagulant prophylaxis [24] or patients with previous arterial thrombosis due to APS [11]. Our registry, in contrast, focuses on this breakthrough thrombosis while on therapeutic anticoagulation and did not explicitly exclude triple-positive patients or patients with arterial events, so called high-risk patients.

The retrospective nature of data collection is one of the main limitations of our registry, as well as the small number of patients, as it is often the case for rare diseases. While analyzing the patients’ medical records, it became obvious that there were missing or inconsistent data and some patients were not able to fill these gaps in knowledge on the phone. Furthermore, patients on DOAC as a long-term medication were selected according to their risk profile and individual preferences in a shared decision-making process. Therefore, there was no unbiased selection of patients in the DOAC arms. Patients were switched from one to another DOAC if they reported increased bleeding, in most cases, heavy menstrual bleeding. From February 2018, following the premature termination of the TRAPS study, patients with triple-positive APS were advised to change rivaroxaban to other anticoagulants and most of them were switched to VKA, which explains the significantly higher proportion of triple-positive-VKA-treated patients at inclusion in the registry (see Table 1). Non-adherence might have been a major reason for the higher recurrence rates of arterial thrombosis in DOAC-treated patients due to the lower half-life time of DOAC in comparison to VKA, but data on non-compliance and non-adherence were difficult to gain. According to Malec et al., it can be hypothesized that failure of DOAC in the management of APS could be, at least in part, related to poor compliance and insufficient knowledge of its therapy [20]. Abdou et al. [25] suggested the following risk factors as relevant to nonadherence: male gender [26], younger age (<60 years), mental health dysfunction [27], certain co-morbidities (depression, alcohol abuse, etc. [28,29]), and side effects of medications [30].

## 5. Conclusions

In summary, according to the results of the APSantiCO registry, APS patients with arterial thrombosis are at particular risk of recurrent arterial thrombosis during therapy with DOAC. This patient group and those with triple positivity should receive VKA, which is in accordance with the recently published Guidance document from the Scientific and Standardization Committee of the International Society on Thrombosis and Haemostasis [31]. In APS patients with venous thrombosis, the results of this registry suggest that physicians can consider DOAC treatment in non-triple-positive APS patients on a case-by-case basis, taking into account the presence of additional risk factors for venous and arterial thrombosis [32] and the risk for bleeding [8]. DOAC treatment may also be considered in the case of contraindications to VKA, medical interactions with VKA, impracticable INR measurement, or problems finding the correct medication dose. Due to the short half-life of DOAC patient’s adherence, persistence is essential in APS patients and intensive patient education is required when using DOAC. Taken together, the potential use of DOACs in APS requires further, appropriately designed, prospective clinical studies.

## Figures and Tables

**Table 1:** Baseline characteristics of patients at inclusion in the registry.

	Study Population	Patients on VKA	Patients on DOAC	*p*-Value Comparing VKA vs. DOAC	Patients on Subcutaneous Anticoagulation and/or Platelet Aggregation Inhibitors
Total number of patients	80	35	29 *	0.532	16
female, % (counts/*n*)	52.5% (42/80)	51.4% (18/35)	41.4% (12/29)	0.460	75% (12/16)
BMI. Median (25–75% Percentiles)	26.3	26.3	25.3	0.672	26.7
	(24.2–29.9)	(24.4–30.1)	(24.1–30.4)		(23.8–29.5)
Age at index thrombosis Median (years) (25–75% Percentiles)					
	44.5	46	50	0.504	35.5
	(30.8–59.3)	(33–60)	(31–66)		(27–43)
**Index event**					
**venous thromboembolism, %; (counts/*n*)**	71.3% (57/80)	68.6% (24/35)	82.8% (24/29)	0.251	56.3% (9/16)
spontaneous venous thromboembolism	73.7% (42/57)	79.2% (19/24)	58.3% (14/24)	0.212	77.8% (7/9)
Deep leg vein thrombosis Pulmonary embolism ◊ Superficial vein thrombosis Atypical venous thrombosis	66.7% (38/57) 26.3% (15/57) 10.5% (6/57) 15.8% (9/57)	75% (18/24) 37.5% (9/24) 8.3% (2/24) 4.2% (1/24)	66.7% (16/24) 20.8% (5/24) 12.5% (3/24) 20.8% (5/24)	0.244	44.4% (4/9) 11.1% (1/9) 11.1% (1/9) 33.3% (3/9)
**arterial thromboembolism, %; (counts/*n*)**	28.8% (23/80)	31.4% (11/35)	17.2% (5/29)	0.251	43.8% (7/16)
Stroke/TIA myocardial infarction peripheral arterial occlusion atypical arterial embolism	52.2% (12/23) 26.1% (6/23) 13% (3/23) 8.7% (2/23)	72.7% (8/11) 18.2% (2/11) 9.1% (1/11) 0% (0/11)	60% (3/5) 20% (1/5) 20% (1/5) 0% (0/5)	1.000	57.1% (4/7) 14.3% (1/7) 14.3% (1/7) 14.3% (1/7)
**Cardiovascular risk factors %; (counts/*n*)**	86.3% (69/80)	91.4% (32/35)	79.3% (23/29)	0.279	87.5% (14/16)
None	13.8% (11/80)	8.6% (3/35)	20.7% (6/29)	0.279	12.5% (2/16)
Hypertension	48.8% (39/80)	51.4% (18/35)	48.3% (14/29)	1.000	43.8% (7/16)
Diabetes	8.8% (7/80)	8.6% (3/35)	10.3% (3/29)	1.000	6.3% (1/16)
Smoking	37.5% (30/80)	31.4% (11/35)	34.5% (10/29)	1.000	56.3% (9/16)
Hypercholesterolemia	52.5% (42/80)	68.6% (24/35)	48.3% (14/29)	0.128	25% (4/16)
elevated Lipoprotein a	13.8% (11/80)	25.7% (9/35)	6.9% (2/29)	0.093	0% (0/16)
atrial fibrillation %; (counts/*n*)	6.3% (5/80)	8.6% (3/35)	3.4% (1/29)	0.620	6.3% (1/16)
**Antiphospholipid antibodies %; (counts/*n*)**					
Lupus anticoagulants	63.8% (51/80)	77.1% (27/35)	48.3% (14/29)	0.021	62.5% (10/16)
Anticardiolipin antibodies	80% (64/80)	82.9% (29/35)	72.4% (21/29)	0.372	87.5% (14/16)
Anti-2 glycoprotein I antibodies	70% (56/80)	80% (28/35)	55.2% (16/29)	0.028	75% (12/16)
Single positivity	27.5% (22/80)	17.1% (6/35)	41.4% (12/29)	0.050	25% (4/16)
Double positivity	31.3% (25/80)	25.7% (9/35)	41.4% (12/29)	0.245	25% (4/16)
Triple positivity	41.3% (33/80)	57.1% (20/35)	17.2% (5/29)	0.002	50% (8/16)
**Autoimmune disease** %; (counts/*n*)	27.5% (22/80)	22.9% (8/35)	27.6% (8/29)	0.774	37.5% (6/16)
**Inherited thrombophilia** %; (counts/*n*) **#**	11.3% (9/80)	2.9% (1/35)	6.9% (2/29)	0.586	37.5% (6/16)

* 29 patients on DOAC: one patient was treated with edoxaban, 25 patients with apixaban, and 3 patients with rivaroxaban; ◊ 4 patients suffered a pulmonary embolism (PE) without leg vein thrombosis, while in 11 patients, the deep leg vein thromboses was accompanied by PE; # G20210A prothrombin mutation, Factor V Leiden mutation, Antithrombin-, protein C- or protein S-deficiency. Direct oral anticoagulants (DOAC), Vitamin K antagonists (VKA).

**Table 2:** Incidence rates of arterial thrombosis per 100 patient years for different treatments together with 95% confidence intervals and, in addition, incidence risk ratios using treatment with VKA as reference.

Treatment Groups	Number of Incidences	Percentage of Incidences with Triple Positive aPL %; (Counts)	Overall Length of Treatment Periods (Months)	Incidence/ 100 Patient Years ^1^	Incidence Risk Ratio ^1^	*p*-Value ^1^
VKA	6	33%; (2/6)	3368	0.4 (0.1–2.4)	Reference	
DOAC	12	67%; (8/12)	1575	4.5 (1.5–13.3)	10.4 (2.8–39.1)	<0.001
Apixaban	3	0%; (0/3)	944	2.4 (0.6–6.6)	3.4 (0.6–17.9)	0.1518
Rivaroxaban	5	80%; (4/5)	525	6.1 (1.6–23.0)	8.5 (1.9–38.5)	0.0058
Edoxaban	2	100%; (2/2)	52	n.a. ^2^		
Dabigatran	2	100%; (2/2)	54			
s.c. anticoagulation ^3^	5	100%; (5/5)	338	6.2 (1.3–29.8)	14.3 (3.0–67.2)	<0.001
Antiplatelet drug	6	0%; (0/6)	2059	1.3 (0.3–5.9)	3.1 (0.7–13.4)	0.1329
s.c. anticoagulation and antiplatelet drug	2	0%; (0/2)	189	2.7 (0.3–24.5)	6.1 (0.8–49.0)	0.0872

aPL antiphospholipid antibodies, n.a. not applicable. ^1^ Rates and ratios are from a mixed effect regression model and do not coincide with raw estimators. ^2^ To avoid extremely wide confidence intervals, incidence rates and incidence risk ratios for treatment groups with less than 100 months observation time were not analyzed. ^3^ Fondaparinux or LMWH or UFH.

**Table 3:** Incidence rates of venous thrombosis per 100 patient years for different treatments together with 95% confidence intervals and, in addition, incidence risk ratios using treatment with VKA as reference.

Treatment Groups	Number of Incidences	Percentage of Incidences with Triple Positive aPL %; (Counts)	Overall Length of Treatment Periods (Months)	Incidence/ 100 Patient Years ^1^	Incidence Risk Ratio ^1^	*p*-Value ^1^
VKA	10	80%; (8/10)	3368	1.6 (0.6–4.5)	Ref.	
DOAC	5	80%; (4/5)	1575	1.5 (0.4–5.1)	0.9 (0.3–3.3)	0.9086
Apixaban	3	67%; (2/3)	944	1.6 (0.4–6.6)	1.0 (0.2–5.2)	0.9634
Rivaroxaban	2	100%; (2/2)	525	1.5 (0.2–12.4)	1.0 (0.1–9.2)	0.9917
Edoxaban	0	0	52	n.a. ^2^		
Dabigatran	0	0	54			
s.c. anticoagulation ^3^	5	40%; (2/5)	338	5.9 (1.4–25.2)	3.7 (1.0–14.5)	0.0581
Antiplatelet drug	13	23%; (3/13)	2059	4.8 (1.9–11.7)	3.0 (1.0–8.8)	0.0471
s.c. anticoagulation and antiplatelet drug	1	100%; (1/1)	189	3.8 (0.4–34.5)	2.4 (0.2–23.1)	0.4530

aPL antiphospholipid antibodies, n.a. not applicable. ^1^ Rates and ratios are from a mixed effect regression model and do not coincide with raw estimators. ^2^ To avoid extremely wide confidence intervals, incidence rates and incidence risk ratios for treatment groups with less than 100 months observation time were not analyzed. ^3^ Fondaparinux or LMWH or UFH.

**Table 4:** Incidence rates of bleedings per 100 patient years for different treatments together with 95% confidence intervals and, in addition, incidence risk ratios using treatment with VKA as reference.

Treatment Groups	Number of Incidences	Overall Length of Treatment Periods (Months)	Incidence/ 100 Patient Years ^1^	Incidence Risk Ratio ^1^	*p*-Value ^1^
VKA	8	3368	1.6 (0.5–4.9)	Reference	
DOAC	8	1575	2.6 (0.7–9.0)	1.6 (0.5–5.1)	0.4536
Apixaban	5	944	2.5 (0.6–10.7)	1.6 (0.4–6.5)	0.4812
Rivaroxaban	2	525	1.4 (0.2–9.3)	0.9 (0.2–5.4)	0.9359
Edoxaban	1	52	n.a. ^2^		
Dabigatran	0	54			
s.c. anticoagulation ^3^	3	338	4.0 (0.7–23.0)	2.4 (0.4–13.3)	0.3053
Antiplatelet drug	2	2059	0.3 (0.0–2.4)	0.2 (0.0–1.2)	0.0733
s.c. anticoagulation and antiplatelet drug	2	189	6.9 (1.1–43.5)	4.3 (0.7–27.4)	0.1276

n.a. not applicable. ^1^ Rates and ratios are from a mixed-effect regression model and do not coincide with raw estimators. ^2^ To avoid extremely wide confidence intervals, incidence rates and incidence risk ratios for treatment groups with less than 100 months observation time were not analyzed. ^3^ Fondaparinux or LMWH or UFH.
